# Spatial Dynamics Links PD-L1 and Tumor-Associated Macrophage-Enriched Niches to Immune and Mesenchymal States in Microsatellite-Stable Colorectal Cancer

**DOI:** 10.3390/cancers18081288

**Published:** 2026-04-18

**Authors:** Brenda Palomar de Lucas, María Ortega, Daniel G. Camblor, Francisco Gimeno-Valiente, Aitana Bolea, David Moro-Valdezate, Jose Francisco González-Muñoz, Marisol Huerta, Susana Roselló, Desamparados Roda, Andrés Cervantes, Noelia Tarazona, Carolina Martínez-Ciarpaglini

**Affiliations:** 1 Department of Pathology, Hospital Clínico Universitario de Valencia, 46010 Valencia, Spain; bpalomar@incliva.es (B.P.d.L.); cortega@incliva.es (M.O.); abolea@incliva.es (A.B.); jgonzalez@incliva.es (J.F.G.-M.); 2 INCLIVA Biomedical Research Institute, 46010 Valencia, Spain; dgonzalez@incliva.es (D.G.C.); fgimeno@incliva.es (F.G.-V.); mhuerta@incliva.es (M.H.); srosello@incliva.es (S.R.); droda@incliva.es (D.R.); andres.cervantes@uv.es (A.C.); noetalla@incliva.es (N.T.); 3 Department of Surgery, University of Valencia, 46010 Valencia, Spain; morp_dav@gva.es; 4 Instituto de Salud Carlos III, CIBERONC, Centro de Investigación Biomedica en Red, 28029 Madrid, Spain; 5 Department of General and Digestive Surgery, Hospital Clínico Universitario de Valencia, 46010 Valencia, Spain; 6 Department of Medical Oncology, Hospital Clínico Universitario de Valencia, 46010 Valencia, Spain

**Keywords:** microsatellite stable, colorectal cancer, PD-L1, tumor-associated macrophages, mesenchymal

## Abstract

**Background/Objectives**: MSS-CRC comprises a heterogeneous group of tumors generally considered “immune cold” due to limited neoantigen generation and T-cell exclusion or inactivation. Current evidence indicates that the composition of T and B immune cells within the tumor microenvironment represents a prognostically relevant factor, significantly associated with both tumor expression profiles and molecular subtypes. **Methods**: We conducted an exploratory analysis to identify prognostically relevant immune cell components in this group of tumors and to investigate corresponding differences in RNA-based bulk expression and high-resolution spatial transcriptomic profiles. **Results**: A total of 254 localized mismatch repair-proficient colorectal cancer cases were evaluated. Our findings revealed PD-L1 expression as a robust independent prognostic biomarker associated with favorable outcomes in this specific population. Bulk RNA expression analysis showed that PD-L1-negative tumors exhibited an expression profile consistent with abundant cancer-associated fibroblast infiltration, increased matrix stiffness, and impaired immune activation—features consistent with tumor progression and poorer clinical outcomes. In contrast, PD-L1-positive tumors displayed stromal programs enriched in immune activation and controlled remodeling, consistent with an immunologically active microenvironment. Spatial transcriptomics added an additional layer of evidence, revealing that epithelial to mesenchymal transition-related programs can dominate stromal niches in PD-L1-negative tumors, particularly within macrophage-enriched stromal regions. **Conclusions**: Our observations suggest an association between PD-L1 expression on immune cells and immune-activated versus mesenchymal-dominant states, potentially occurring within macrophage-enriched stromal niches. These results provide insight into the biological mechanisms underlying disease progression and highlight tumor-associated macrophages as a potential therapeutic target to overcome immune resistance, particularly in PD-L1-negative MSS-CRC tumors.

## Simple Summary

Colorectal cancer is among the most common malignancies worldwide. Approximately 85% of cases are classified as microsatellite-stable colorectal cancer (MSS-CRC), a subgroup considered immunologically “cold,” characterized by limited immune cell infiltration and a poor response to immunotherapy. We analyzed tumor tissue from 254 patients with MSS-CRC to identify prognostically relevant features of the tumor microenvironment. PD-L1 expression on immune cells was strongly associated with prolonged disease-free survival, independent of other clinical factors. Bulk transcriptomic analysis revealed that PD-L1-negative tumors exhibited molecular features consistent with mesenchymal transition, whereas PD-L1-positive tumors displayed an immune-activated phenotype. Using high-resolution spatial transcriptomics, we observed that these differences localized specifically to tumor-associated macrophage-enriched niches. Our findings suggest that tumor-associated macrophages exhibit fundamentally distinct immune and molecular landscapes depending on PD-L1 expression status, highlighting a potential avenue for the development of novel therapeutic strategies in this challenging colorectal cancer subtype.

## 1. Introduction

Colorectal cancer (CRC) remains the most common gastrointestinal cancer and the third leading cause of cancer-related death in both men and women [1]. Although early-onset (new diagnosis in individuals <50 years) CRC incidence rates are rising, the overall prognosis has improved considerably over the past two decades, largely due to advances in surgical and clinical management, the development of novel therapeutic agents, refinement of patient selection, and early detection through screening programs [2,3,4,5]. The recognition of microsatellite instability (MSI) as a key prognostic and predictive biomarker in both localized and metastatic CRC has also contributed significantly to this trend [6]. MSI-positive CRC is highly immunogenic, characterized by strong tumor-specific immune activation, and demonstrates substantial clinical benefit from treatment with immune checkpoint inhibitors (ICIs) [7]. However, most CRC cases (80–85% localized and <5% metastatic) show no significant alterations in microsatellite length (microsatellite stable, MSS). These tumors are generally considered “immune cold” due to limited neoantigen generation and T-cell exclusion or inactivation [8]. Nevertheless, MSS CRC represents a heterogeneous group of tumors, and novel drug combinations—including immunotherapy—have thus far yielded only modest clinical efficacy [8,9].

In 2006, Galon et al. demonstrated that the type, location, and density of immune T-cells within the tumor microenvironment (TME) are strong predictors of patient survival in CRC, independent of MSI status [10,11,12]. An activated B-cell response also plays a significant role in CRC progression, facilitating the generation of effector memory B-cells and antibody-producing plasma cells within tertiary lymphoid structures (TLSs) [13]. Bulk transcriptomic analyses led to the identification of four consensus molecular subtypes (CMSs) that differ in molecular profile and clinical outcomes, each of them associated with distinct immune signatures [14]. More recent advances in single-cell technologies have further revealed intricate crosstalk among several immune cell types, tumor cells and other components of the TME contributing to cancer progression and immune evasion in CRC [15,16,17,18].

Based on these insights, we designed an exploratory analysis to uncover prognostically relevant TME features in MSS CRC and to investigate corresponding differences in bulk RNA-based expression and high-definition spatial transcriptomics profiles. Gaining deeper insights into the TME composition may be critical for uncovering targetable pathways to overcome immune evasion in this challenging tumor subset.

## 2. Materials and Methods

### 2.1. Patient Cohorts

**Figure A1:**
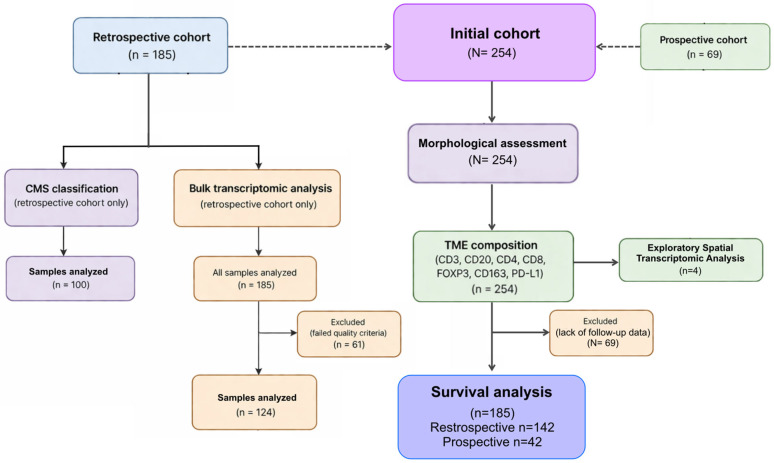
Flowchart illustrating cohort distribution across each section of the study.

Two cohorts of patients with localized mismatch repair-proficient (MMRp) CRC who underwent curative-intent surgery at the Hospital Clínico Universitario of Valencia (Spain) were analyzed. The first cohort, collected retrospectively, included all cases diagnosed between January 2015 and December 2017. The second cohort was prospectively recruited between March 2021 and December 2023 (Table 1). All cases with loss of expression of any mismatch repair (MMR) protein detected by immunohistochemistry (IHC) were excluded from the analysis. For each case, 2–3 formalin-fixed, paraffin-embedded (FFPE) representative tissue blocks were collected. Clinical and follow-up data were retrieved from medical records. Ethical approval was granted by the INCLIVA Health Research Institute Ethics Committee. No formal comparison of clinical or pathological features between cohorts was performed, as both cohorts were analyzed together to increase statistical power for survival analysis and immunohistochemical characterization of the tumor microenvironment. A flowchart illustrating cohort distribution across each section of the study is presented in Appendix Figure A1.

### 2.2. Histological Evaluation

#### 2.2.1. Morphological Assessment

All hematoxylin and eosin (HE)-stained sections from each case were reviewed to assess histological differentiation, lymphovascular invasion (LVI), perineural invasion (PNI), depth of invasion (pT) and number of involved lymph nodes (pN). Tumor budding (TB) and tumor stroma (TS) were analyzed as previously described [19].

#### 2.2.2. Immune Infiltrate Assessment

Quantitative assessment of the TME composition was performed by IHC. All IHC procedures were carried out on an automated platform (Autostainer Link 48, Agilent Technologies, Glostrup, Denmark). The following monoclonal antibodies were used: CD3 (Dako, Glostrup, Denmark, IR503, Ready-to-Use, high pH), CD4 clone 4B12 (Dako, IR649, Ready-to-Use, high pH), CD8 clone C8/144B (Dako, IR623, Ready-to-Use, high pH), CD20cy clone L26 (Dako, IR604, Ready-to-Use, high pH), CD23 clone DAK-CD23 (Dako, IR781, Ready-to-Use, high pH), CD163 (Cell Signaling Technology, Danvers, MA, D6U1J, 1:400 dilution, high pH), FoxP3 (Cell Signaling Technology, D2W8E, 1:100 dilution, high pH), and PD-L1 clone 22C3 (Dako, SK006, Ready-to-Use, low pH).

Slides were digitized at 20× magnification (3DHistech P250, Budapest, Hungary). Positive immune cells were quantified using QuPath (v4.0.0) [20] with a 1.5 µm cell expansion and a 0.15 positivity threshold. Three circular regions (800 µm × 800 µm) with the highest density of positive cells were manually annotated at both the invasive front (IF) and tumor center (TC), an approach demonstrated to be reliable and reproducible for several tumor types [21,22]. CD163-positive macrophages were quantified manually at the IF and TC in one 20X hotspot microscopic field. Immune markers were analyzed both as continuous variables and dichotomized using the mean value due to the lack of established thresholds.

#### 2.2.3. Tertiary Lymphoid Structures

TLSs were defined as nodular aggregates of ≥50 lymphocytes located at the IF in HE-stained sections or CD20 IHC. TLSs were manually quantified in a 1 mm^2^ area. CD23 staining was used to classify TLSs containing follicular dendritic cells (FDCs) as mature (mTLS) and those lacking FDCs as immature (iTLS) [23]. CD20-positive cell density was also measured using QuPath (v4.0.0) across the IF, including TLSs within 7 mm of the IF [20]. TLS density and CD20-positive B-cell infiltration were categorized into high and low based on the mean value.

#### 2.2.4. PD-L1 Assessment

PD-L1 expression was evaluated visually as the percentage of positive immune cells (lymphocytes and macrophages) across the tumor area. Cases presenting staining in >1% immune cells were considered as PD-L1 positive. This threshold was selected after evaluating multiple cut-off values (1%, 5%, and 10%) based on their association with disease-free survival, with only the 1% threshold showing a significant prognostic impact. The percentage of tumor cells showing membranous PD-L1 expression was recorded separately.

### 2.3. Transcriptomic Analysis

#### 2.3.1. RNA Extraction

Representative tumor tissue was macrodissected from FFPE blocks. Total RNA was extracted using the RNeasy FFPE Kit (Qiagen) on the automated Qiacube Connect system, eluted in 30 µL RNase-free water, and assessed for quality and concentration using TapeStation (Agilent). Samples were stored at –80 °C.

#### 2.3.2. CMS Subtype Analysis

Consensus molecular subtypes (CMSs) were determined for 100 tumor samples in the retrospectively collected cohort by using a custom 38-gene NanoCRCA assay in a NanoString nCounter^®^ platform (NanoString Technologies, Bothell, DC, USA) as previously described [24].

#### 2.3.3. mRNA Sequencing

Libraries were prepared and sequenced by Macrogen Inc. (Seoul, Republic of Korea) using the Illumina NovaSeq platform. Raw FastQ files were aligned to the human reference genome (GRCh38), and gene expression levels were quantified using standard bioinformatic pipelines. Differential gene expression analysis was performed using DESeq2 in R (v4.3.0), with *p*-values adjusted for multiple testing using the Benjamini–Hochberg false discovery rate (FDR) method. Statistical significance was defined as an adjusted *p*-value < 0.05. Functional enrichment analysis was conducted using overrepresentation analysis (ORA) with the MSigDB Hallmark gene sets. The epithelial to mesenchymal transition (EMT) score R package was used to evaluate EMT-related gene signatures [25]. EMT score assigns a score based on epithelial and mesenchymal gene expression, allowing classification into epithelial or mesenchymal phenotypes. Transcriptomic analyses were performed exclusively in the retrospective cohort.

#### 2.3.4. Spatial Transcriptomics

Spatial transcriptomics was performed using the Visium HD Spatial Gene Expression platform (10× Genomics, Pleasanton, CA, USA). Four representative cases (two PD-L1 positive, two PD-L1 negative) were selected. FFPE blocks were sectioned (5 µm), hybridized with probes, captured via CytAssist, and libraries constructed using the Visium HD Spatial Gene Expression for FFPE Kit. Libraries were sequenced at Macrogen Inc. (150 bp paired-end reads). Raw FastQ data were processed with the Space Ranger pipeline, aligning reads to GRCh38 and the probe-specific reference. The workflow included barcode and UMI extraction, error correction, spatial binning (8 µm × 8 µm), and tissue barcode filtering. HE reference images were aligned to CytAssist images using fiducial markers. Clusters were automatically generated via dimensionality reduction and supervised clustering visualized in Loupe Browser (v8.1.2). Macrophage-associated clusters (CD163, CD68, CD14, F3, LYZ) and stromal clusters were selected, and cluster-specific gene expression was compared between PD-L1 positive and negative samples using Seurat R package (v4.4.0) [26].

#### 2.3.5. Functional Analysis of Gene Interactions

To explore functional interactions among differentially expressed genes, a network analysis was performed using STRING (Search Tool for the Retrieval of Interacting Genes/Proteins) version 12.0 [27,28]. The list of overexpressed genes obtained from spatial transcriptomics was used, with “Homo sapiens” as the reference organism. Default parameters were applied (“full STRING network”) with a medium confidence threshold of 0.4. The generated network represents known and predicted interactions among the proteins encoded by the genes of interest, including direct and indirect associations based on co-expression, databases, the literature, and bioinformatic predictions. The network was exported as an image for visualization and interpretation, allowing the identification of highly connected functional clusters.

### 2.4. Statistical Analysis

All statistical analyses were performed using R software (v4.3.0, R Core Team, Vienna, Austria). A significance threshold of *p* ≤ 0.05 was applied for all tests [24].

#### 2.4.1. Descriptive and Association Analyses

A comprehensive statistical analysis was conducted for clinical, histological, immunohistochemical, and transcriptomic variables. Associations between categorical variables were assessed using Pearson’s chi-square test (χ^2^). Odds ratios (ORs) with 95% confidence intervals (CIs) were calculated using binary logistic regression. Correlations between numerical variables were assessed according to their distribution. Normality was tested using the Shapiro–Wilk test. If normality was rejected (*p* < 0.05), non-parametric tests were applied, including the Wilcoxon rank-sum test for two-group comparisons and the Kruskal–Wallis test for comparisons involving more than two groups. For normally distributed data, homogeneity of variances was evaluated using Levene’s test, followed by the independent samples Student’s *t*-test, as appropriate. Associations between quantitative variables were assessed using Spearman’s rank correlation coefficient, with values close to 1 or –1 indicating strong positive or negative correlations, respectively.

#### 2.4.2. Survival Analysis

Disease-free survival (DFS) was defined as the time from curative-intent colectomy to the occurrence of the first relapse or metastasis. Survival functions were compared between groups using the log-rank test. DFS was visualized using Kaplan–Meier curves, and *p*-values were calculated with the χ^2^ test to assess differences between curves. Hazard ratios (HRs) and 95% confidence intervals (CIs) were estimated using the Cox proportional hazards model. Multivariable Cox regression models were constructed, including variables with clinical relevance and/or statistical significance in univariable analysis to identify independent predictors. Collinearity among variables was assessed based on clinical relevance and correlation between variables. The proportional hazards assumption was assessed using Schoenfeld residuals. HRs are reported with 95% CIs. Survival analyses were restricted to cases with complete follow-up data. Missing data were handled using complete-case analysis.

## 3. Results

### 3.1. General Clinicopathological Features

A total of 254 cases of localized MMRp CRC were evaluated, of which 185 were collected retrospectively and 69 prospectively (Table 1). The mean age at diagnosis was 65 years (range 37–82), and the sex distribution was balanced. Most tumors were in the left colon (58.3%), followed by the right colon (29.9%) and rectum (11.8%). Classical adverse pathological features, such as LVI, PNI, and poor differentiation, were observed in 27.2%, 18.9%, and 21.3% of cases, respectively.

Although MMR-deficient cases were excluded during case selection, 15 (15%) of the cases with available CMS data were still classified as CMS1. Cases classified as CMS1 were re-evaluated, confirming preserved MMR protein expression in all instances. In addition, all cases were classified as MSS through PCR (Idylla™, Biocartis, Mechelen, Belgium).

### 3.2. Tumor Microenvironment Composition: TLS-Enriched Tumors Are Characterized by a Pro-Inflammatory Immune Microenvironment

**Figure 1:**
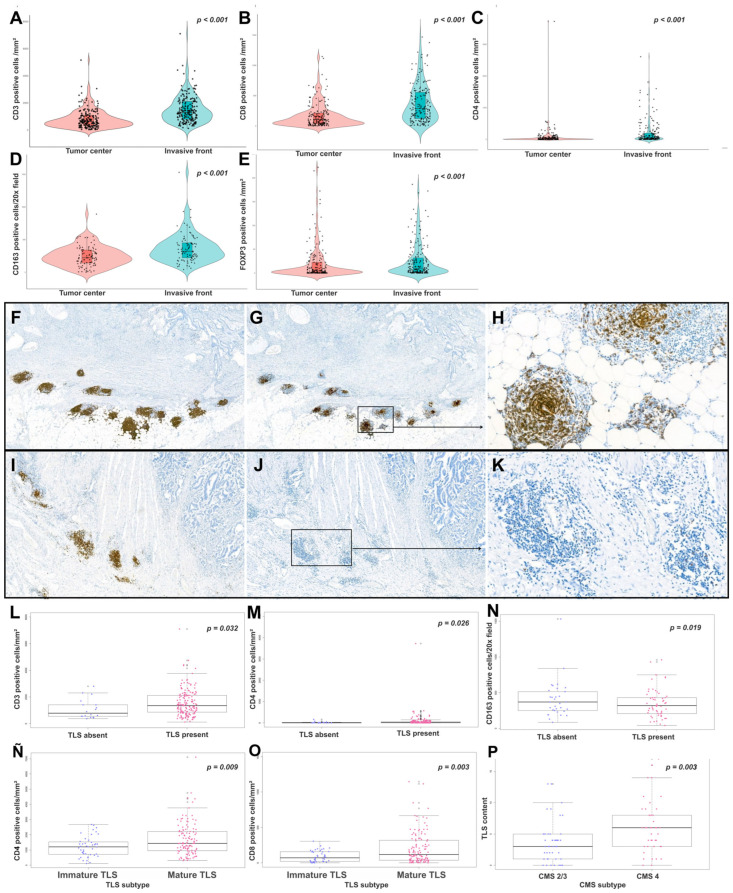
Immune cell densities and tertiary lymphoid structures in MSS CRC. (**A–E**) CD3^+^, CD4^+^, CD8^+^, CD163^+^, and FoxP3^+^ immune cells were significantly more abundant at the invasive front (IF) than at the tumor center (TC). (**F**) Representative case showing high CD20^+^ TLS content (CD20, 4×), (**G**,**H**) most of which contained CD23^+^ follicular dendritic cell networks categorized as mature TLSs (mTLSs) (CD23, 4× and 20×, respectively). (**I**) Representative case showing high CD20^+^ TLS content (CD20, 4×), (**J**,**K**) with complete absence of CD23^+^ dendritic cells, categorized as immature TLSs (iTLSs) (CD23, 4× and 20×, respectively). (**L**–**P**) TLS-positive tumors showed higher CD3^+^ (**L**) and CD4+ (M) T-cell densities and lower CD163^+^ (**N**) macrophage density at the IF. Mature TLSs were associated with increased CD4^+^ (**Ñ**) and CD8^+^ (**O**) T-cell infiltration compared with immature TLSs. TLS content was highest in mesenchymal CMS4 cases compared with CMS2/3 subtypes (**P**). (Wilcoxon rank-sum test).

The TME composition was successfully analyzed in the entire series (n = 254). The density of CD3^+^, CD4^+^, CD8^+^, CD163^+^, and FoxP3^+^ immune cells was significantly higher at the IF compared with the TC (all *p* < 0.001) (Figure 1A–E). A strong positive association was found between CD3 and CD8 cell densities at the IF (Spearman’s ρ = 0.61, *p* < 0.001). No significant associations were observed between immune cell densities and adverse histopathological features.

TLSs were identified in 71.6% of cases using HE and in 90.3% of cases by CD20 IHC, with a moderate correlation between the two methods (ρ = 0.44, *p* < 0.001). By IHC, 43.2% of cases were classified as TLS-high versus 37.7% by HE. Most cases (66%) presented mTLS, characterized by the presence of CD23^+^ follicular dendritic cells (Figure 1F–K).

CD20^+^ B-cell density at the invasive front (IF) was high in 49.7% of cases and was strongly associated with the number of TLSs (ρ = 0.74, *p* < 0.001). Tumors with TLSs were significantly enriched in CD3^+^ T-cells at the IF (mean 1348.8 vs. 780.5 cells/mm^2^, *p* = 0.032) (Figure 1L). A total of 78.4% of TLS-positive cases had high CD3 density compared to 50% of TLS-negative cases (*p* = 0.007). TLS presence was also associated with higher CD4^+^ T-cell density at the TC (12.6 vs. 1.3 cells/mm^2^, *p* = 0.026) (Figure 1M); a similar pattern was observed at the IF (*p* = 0.052). Conversely, macrophage density (CD163^+^) was significantly lower at the IF in TLS-positive tumors (310 vs. 517.5 cells/mm^2^, *p* = 0.019) (Figure 1N). TLS-negative tumors were more frequently associated with high CD163^+^ density (87.5% vs. 48.8%, *p* = 0.036).

Mature TLSs were linked to significantly higher infiltration of CD3^+^ T-cells at the IF (53.05 vs. 20.22 cells/mm^2^, *p* = 0.009) and increased CD8+ T-cell density at both the TC and IF (*p* = 0.003 and *p* = 0.022, respectively) compared to iTLSs (Figure 1Ñ,O). Mesenchymal CMS4 cases showed the highest TLS content compared with CMS2/3 subtypes, both in analyses based on HE (*p* = 0.020) and CD20 IHC (*p* = 0.003) (Figure 1P).

### 3.3. PD-L1 Expression in Immune Cells Is Associated with Improved Disease-Free Survival Independent of Clinical Stage

**Figure 2:**
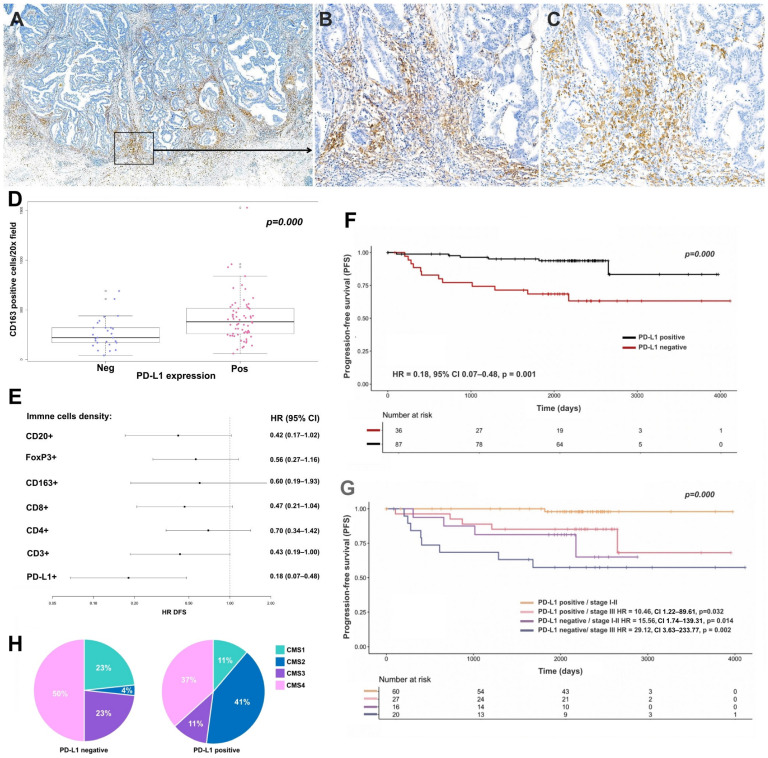
Prognostic impact of PD-L1 expression in MSS colorectal cancer. (**A**,**B**) PD-L1 expression was mainly observed within lymphoid aggregates at the invasive front (PD-L1, 4× and 20×, respectively), (**C**,**D**) and was associated with high CD163^+^ macrophage density (C, CD163, 20×). (**E**,**F**) PD-L1 expression in immune cells was the only tumor microenvironment feature significantly associated with significantly improved DFS. (**G**) Combined PD-L1 immune expression and clinical stage stratification identified four prognostic groups. (**H**) PD-L1-negative tumors were enriched in the CMS4 subtype.

**Figure A2:**
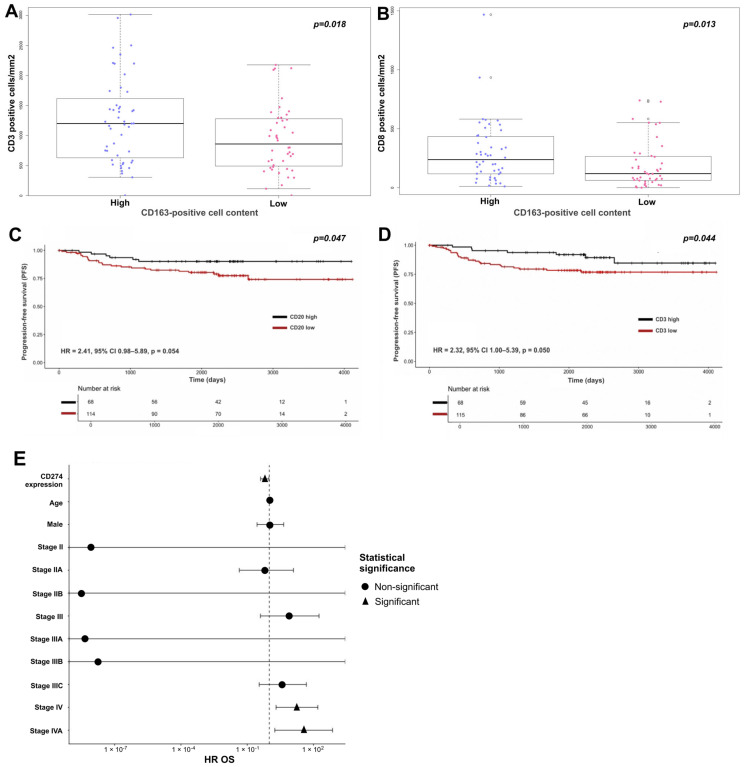
(**A**,**B**) CD163 density was positively associated with the content of CD3 and CD8-positive cells at the invasive front. (**C**,**D**) CD3^+^ and CD20^+^ cell densities presented only borderline prognostic value. (**E**) An exploratory analysis of MSS colorectal cancer patients in the TCGA dataset showed that PD-L1 mRNA (CD274) expression was significantly associated with improved overall survival probability.

PD-L1 expression was assessed in the entire cohort (n = 254). Tumor cell PD-L1 positivity was infrequently observed (n = 7; 3%). In contrast, PD-L1 expression in immune cells was observed in 70% of cases, predominantly within the lymphoid infiltrate at the tumor IF (Figure 2A,B). PD-L1-positive cases showed higher CD163^+^ macrophage density at the IF (mean 380 vs. 220 cells/mm^2^, *p* = 0.001). High CD163 density was associated with increased odds of PD-L1 positivity (OR = 4.92, 95% CI 2.14–11.28, *p* < 0.001), whereas low CD163 density was associated with reduced odds (OR = 0.37, 95% CI 0.13–0.96, *p* = 0.049) (Figure 2C,D). CD163 density was also positively associated with CD3^+^ and CD8^+^ cell densities at the invasive front (Spearman’s ρ, *p* = 0.018 and *p* = 0.013, respectively; Appendix Figure A2A,B).

Survival analyses were performed in cases with available follow-up data (n = 185), including 42 from the prospective cohort and 142 from the retrospective cohort (mean follow-up: 66 months). Cases lacking follow-up information were excluded from survival analyses. A total of 31 events were observed. In univariable analysis, immune cell PD-L1 expression was significantly associated with early clinical stage and absence of adverse prognostic factors (Table 2). PD-L1 expression was the only TME variable significantly associated with DFS (Figure 2E). Patients with PD-L1-positive immune cells had improved DFS compared with PD-L1-negative cases (HR = 0.18, 95% CI 0.07–0.48, *p* = 0.001; 5-year DFS: 73% vs. 52%, log-rank *p* < 0.001) (Figure 2F). Among the evaluated thresholds, the 1% cut-off was the only one significantly associated with DFS. Low CD3^+^ T-cell density in the TC and low CD20^+^ B-cell density showed only borderline associations with increased risk of recurrence (CD3 in TC: HR = 2.32, 95% CI 1.00–5.39, *p* = 0.050; CD20: HR = 2.41, 95% CI 0.98–5.89, *p* = 0.054) (Appendix Figure A2C,D). Lymph node invasion was significantly associated with DFS (HR = 4.52, 95% CI 2.09–9.78, *p* < 0.001). PD-L1 expression in tumor cells showed no association with prognosis (*p* = 0.998).

No other clinical or histological variables were associated with prognosis. In multivariable analysis, both lymph node involvement (HR = 3.23, 95% CI 1.14–9.31, *p* = 0.028) and PD-L1 expression (HR = 0.24, 95% CI 0.08–0.66, *p* = 0.006) remained independently associated with DFS.

Finally, we explored the clinical value of the combination of PD-L1 expression in immune cells (positive/negative) and the clinical stage into one stratification system feasible to implement in clinical practice. Patients were stratified into four groups: PD-L1-positive stage I–II, PD-L1-positive stage III, PD-L1-negative stage I–II, and PD-L1-negative stage III. This combined stratification was significantly associated with DFS. Compared with PD-L1-positive stage I–II cases (reference), the risk of recurrence increased progressively across groups (PD-L1-positive stage III: HR = 10.46, 95% CI 1.22–89.61, *p* = 0.032; PD-L1-negative stage I–II: HR = 15.56, 95% CI 1.74–139.31, *p* = 0.014; PD-L1-negative stage III: HR = 29.12, 95% CI 3.63–233.77, *p* = 0.002) (Figure 2G). Interestingly, negative PD-L1 expression was significantly associated with the worst prognosis subgroup defined by the tumor budding–stromal content (TBS) score, previously published by our group [19].

Importantly, in multivariable analysis including clinical stage and classical pathological features (LVI, PNI, pT, pN and TB), immune cell PD-L1 positivity remained independently associated with improved disease-free survival (HR 0.45, 95% CI 0.27–0.74, *p* = 0.002). No evidence of problematic collinearity was identified among variables included in the multivariable model. Considering these findings, we explored the TCGA dataset to assess the association between PD-L1 mRNA (CD274) expression and prognosis, restricting the analysis to MSS patients using the R package TCGAbiolinks [29]. In a multivariable Cox model, CD274 expression was significantly associated with improved overall survival (HR = 0.62; 95% CI: 0.40–0.96; *p* = 0.031; Appendix Figure A2E). However, no significant association was observed with disease-free survival (DFS).

PD-L1-negative tumors were also more frequently classified as CMS4 (50%), whereas PD-L1-positive tumors were enriched in CMS2 (41%; *p* = 0.002; Figure 2H).

### 3.4. Bulk Transcriptomic Profiling Reveals Mesenchymal and Immune-Activated Profiles According to PD-L1 Status

Bulk transcriptomic analysis was conducted on all cases from the retrospective cohort (n = 185); however, only 124 samples met the predefined quality criteria and were included in the final analysis.

**Figure 3:**
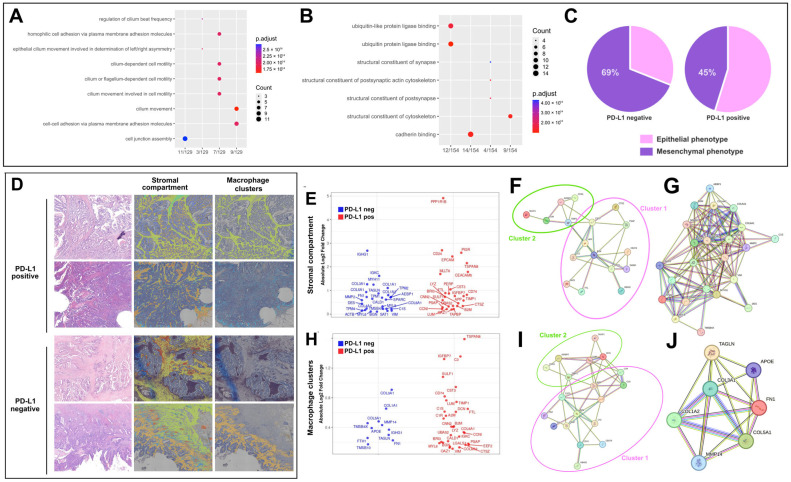
Transcriptomic characterization of PD-L1–positive and PD-L1 negative MSS colorectal cancers. (**A**) Bulk RNA–seq analysis revealed an overrepresentation of cell junction and motility processes in PD-L1–positive cases. (**B**) PD-L1–negative tumors presented overrepresentation of molecular functions related to structural constituents of the cytoskeleton and cadherin binding. (**C**) Logistic regression using the EMT score package showed that mesenchymal tumors were predominantly PD-L1–negative. (**D**) Spatial transcriptomics identified multiple transcriptionally distinct clusters; stromal and macrophage-rich regions were selected for comparison. (**E**) PD-L1–positive tumors significantly overexpressed innate immunity and antigen–presentation genes, whereas PD-L1–negative tumors upregulated extracellular matrix and contractile genes. (**F**) STRING analysis identified two interconnected functional clusters related to immune activation (pink ellipse) and tissue remodeling (green ellipse) in PD-L1–positive tumors (**G**) versus structural and ECM modules in PD-L1-negative cases. (**H**) The macrophage–rich stroma showed a distinct expression profile according to PD-L1 status. (**I**) PD-L1–positive cases exhibited significant overexpression of genes related to strong immune activity (pink ellipse) and tissue remodeling (green ellipse), forming two closely interconnected clusters in the STRING analysis, (**J**) whereas PD-L1-negative counterparts displayed a functional cluster of genes associated with a mesenchymal, CAF-like profile.

Bulk RNA expression profiles were analyzed based on PD-L1 IHC expression in immune cells. PD-L1 immune-positive tumors exhibited significant upregulation of genes involved in immune response, including lectin-type receptors (CLEC18A/B/C), inflammatory molecules (CASP5, IL31RA), and adhesion-related genes (ITGA10) [30]. These cases showed overrepresentation of cell adhesion and cilium movement-related processes (Figure 3A). Additionally, molecular function enrichment revealed upregulated pathways associated with ion transport and synaptic signaling, suggesting enhanced immune and intercellular communication activity in PD-L1-positive tumors [30,31,32,33,34,35,36]. In contrast, PD-L1-negative tumors were enriched in genes related to cell adhesion, cytoskeletal organization, and invasiveness, including ADAM15, THBS1, FLNA, ACTG1, TUBA1C and PKM, which have been previously linked to EMT [37,38], cell motility and poor prognosis [38,39]. Gene ontology enrichment revealed overrepresentation of molecular functions related to cell structural support, particularly related to “structural constituents of the cytoskeleton”, “cadherin binding” and “structural constituents of synapses” (Figure 3B). These findings suggest that PD-L1-negative cases are more closely associated with a transcriptional program favoring mesenchymal plasticity and structural remodeling of the tumor microenvironment.

Using the EMT score package, logistic regression analysis revealed that mesenchymal tumors were more frequently PD-L1 negative compared with epithelial tumors. Notably, 69% of PD-L1-negative cases exhibited a mesenchymal profile (OR = 0.37, *p* = 0.033, Figure 3C).

### 3.5. Spatial Transcriptomics Suggests Compartment-Specific Stromal and Macrophage-Enriched Niches Linked to EMT–Immune Interactions According to PD-L1 Status

To achieve spatial resolution, we applied spatial transcriptomics (Visium HD, 10× Genomics) in four representative cases (two PD-L1 positive, two PD-L1 negative) selected from the retrospective cohort, all corresponding to stage II disease. Using Loupe Browser, multiple transcriptionally distinct clusters were automatically identified in each sample. To perform a comparative analysis, we selected stromal regions and, separately, the cluster with the highest expression of macrophage-associated genes, based on the strong link between PD-L1 and CD163 described previously (Figure 3D).

#### 3.5.1. Spatial Transcriptomic Profile of the Stromal Compartment

In the stromal compartment, clear transcriptomic differences were observed between PD-L1 positive and PD-L1-negative cases. PD-L1-positive tumors showed overexpression of genes associated with innate immunity and antigen presentation (e.g., LYZ, CST3, CD74, B2M, TAPBP), along with tissue remodeling-related genes (IGFBP7, TIMP1) [40,41,42,43,44,45]. In contrast, PD-L1-negative cases showed predominant overexpression of genes related to the extracellular matrix (COL1A1, COL3A1, COL5A1, COL6A1, COL6A2), cytoskeletal components, actin, and myofilaments (FN1, VIM, TAGLN, MYH11, ACTB, CALD1, DES) [30,31,32,33,34,35,36,46], as well as remodeling enzymes such as metalloproteinases (MMP2) [47] (Figure 3E).

STRING network analysis of the most significantly overexpressed genes in PD-L1-positive cases identified two well-connected functional clusters associated with the core processes of immune response, antigen processing, and tissue remodeling that shape an immunologically active tumor microenvironment (Figure 3F). Cluster 1 (immune response and antigen presentation; pink ellipse) included CD74, B2M, TAPBP, APP, LYZ, CST3, PSAP, CTSZ, and others. These genes are pivotal for antigen presentation, inflammation, and immune activation. Notably, CD74 regulates MHC class II antigen presentation and interacts with MIF, influencing PD-L1 expression. B2M and TAPBP are essential for MHC class I peptide presentation, while LYZ and CST3 contribute to innate immune defense and regulation of tumor cell migration [48,49,50,51]. Cluster 2 (tissue remodeling and response; green ellipse) included TIMP1, IGFBP7, LUM, CCN1 and SULF1. These genes are linked to ECM remodeling, angiogenesis, and cell adhesion. TIMP1 not only inhibits metalloproteinases but also promotes immune infiltration by macrophages and neutrophils, while IGFBP7 has been associated with tumor suppression and EMT inhibition in CRC [43,44].

In contrast, using STRING network analysis, PD-L1-negative tumors overexpressed genes related to ECM components (COL1A1, COL3A1, COL5A1, COL6A1, COL6A2) [52], cytoskeletal and contractile elements (FN1, VIM, ACTA2, TAGLN, MYH11, CALD1, DES) [28,38,53,54], and remodeling enzymes (MMP2) (Figure 3G) [39]. Importantly, FN1, VIM, and MMP2 are classical mesenchymal markers [55], reinforcing the mesenchymal phenotype observed in PD-L1-negative cases. STRING network analysis highlighted a robust interaction cluster dominated by ECM and cytoskeletal genes, reflecting a contractile and reactive stroma.

#### 3.5.2. Spatial Transcriptomic Profile of Macrophage-Rich Stromal Clusters

This analysis showed that the macrophage-rich stromal cluster was enriched in immune-related genes, with PD-L1-positive tumors showing marked overexpression of LYZ, CD74, and CST3, all previously linked to an immunoreactive phenotype (Figure 3H) [40,41,42,43].

STRING network analysis of overexpressed genes in PD-L1-positive cases again revealed two well-connected functional clusters related to a more robust immune profile, incorporating additional genes involved in inflammation (C3, A2M, C1R, C1S) and stromal modulation (Figure 3I). Complement 3 (C3) is a central member of the complement system, capable of promoting macrophage and dendritic cell recruitment, enhancing phagocytosis, and contributing to antitumor innate immune responses [56]. A2M (alpha-2-macroglobulin) is a protease inhibitor and cytokine carrier that can regulate inflammation by sequestering pro-inflammatory mediators [57]. C1R/C1S subunits of the C1 complex initiate the classical complement pathway, facilitating phagocytosis, inflammation, and cell lysis [58]. DCN (Decorin) is an extracellular matrix proteoglycan with structural and immunomodulatory functions, able to inhibit TGF-β, stimulate autophagy, and modulate angiogenesis and tumor progression [59].

By contrast, the equivalent macrophage-rich stromal clusters in PD-L1-negative tumors showed a fundamentally different profile (Figure 3J): despite high macrophage content, these clusters predominantly overexpressed extracellular matrix structural genes (COL1A1, COL5A1, COL6A1), remodeling enzymes (MMP14), and classical cancer-associated fibroblast (CAF) markers (FN1, TAGLN), without evidence of immune-related transcriptional activity [39,52,53,54,60,61]. The STRING network identified a tightly connected structural and contractile stromal module, characteristic of an active mesenchymal phenotype.

## 4. Discussion

We performed an integrative analysis of MSS CRC cases at localized stages focusing on classical morphological prognostic features, TME composition and RNA-based bulk and high-definition spatial expression profiles.

According to our findings, immune PD-L1 expression in MSS CRC emerged as a robust, independent prognostic biomarker, associated with the absence of histopathological risk factors and favorable prognosis in this specific population. PD-L1 positivity was most frequently observed in early clinical stages, whereas cases with lymph node metastasis were more often PD-L1 negative. Despite this association, PD-L1 remained significantly linked with prognosis independent of pN status. Furthermore, combining PD-L1 immune status (positive/negative) with clinical stage (I-II/III) showed that PD-L1-negative tumors, even at early stages (I–II), exhibited significantly shorter DFS than PD-L1-positive tumors, even at stage III. Our exploratory analysis of the TCGA dataset also suggests a favorable association between CD274 mRNA expression and prognosis among MSS CRC patients. Nevertheless, CD274 mRNA expression is highly variable and context-dependent and may not fully recapitulate immune cell PD-L1 expression as assessed by immunohistochemistry [62].

Independently published series have reported inconsistent association between PD-L1 status and clinical outcomes [63,64,65]. These discrepancies may be explained by the inclusion of both MSI and MSS patients within the same analyses, as MSI-CRC is typically associated with high PD-L1 expression [66]. In addition, differences in the cellular source of PD-L1 expression (tumor cells versus immune cells) and the antibody clones used for detection may further contribute to the variability in reported results [63]. The positive association observed between PD-L1 expression and survival may appear paradoxical, given that the primary function of PD-L1 is to suppress T-cell-mediated immune responses through its interaction with PD-1 (programmed cell death protein 1). However, substantial evidence supports PD-L1 expression in immune cells and tumor cells, carrying divergent prognostic implications. PD-L1 expression on immune cells is generally associated with an activated inflammatory tumor microenvironment, often induced by interferon-γ (IFN-γ), a key pro-inflammatory cytokine [67]. Consistent with this, in two independent localized CRC series, PD-L1 expression has been found to be independently associated with improved survival only when assessed on immune cells, whereas PD-L1 expression on tumor cells was infrequent and showed no prognostic association, aligning with our observations [68,69]. Additionally, multiple studies have shown that PD-L1 expression is closely associated with increased immune cell infiltration [70,71].

In our series, 50% of PD-L1-negative cases were classified as mesenchymal CMS4 molecular subtype and 69% presented a mesenchymal phenotype following the EMT score on bulk RNA transcriptomic analysis. PD-L1-negative tumors displayed an expression profile consistent with abundant CAF infiltration, increased matrix stiffness, and impaired immune activation, aligning with worse outcomes. EMT is a key biological process that promotes MSS CRC progression by facilitating the conversion of epithelial cells into motile mesenchymal phenotypes [72]. Emerging evidence suggests that EMT is intricately linked to multiple tumor hallmarks, including remodeling of the tumor microenvironment through reshaping of stromal and immune cell interactions, thereby contributing to an immunosuppressive state [73,74,75]. Furthermore, histological features indicative of mesenchymal transition—namely TB and TS—are linked to reduced immune infiltration and adverse prognosis [75,76,77].

Our spatial transcriptomic analysis prioritized stromal and macrophage niches, based on the strong association between immune PD-L1 expression and mesenchymal phenotype and by the enrichment of CD163^+^ tumor-associated macrophage density in PD-L1-positive cases. This approach is further supported by consistent evidence across multiple tumor types, indicating that tumor-associated macrophages (TAMs) are the predominant immune cell population expressing PD-L1 within the stromal compartment [78,79]. Importantly, using clustering analysis, spatial transcriptomics added a novel layer of evidence, revealing that EMT-related programs can dominate stromal niches in PD-L1-negative tumors, specifically in macrophage-enriched stromal areas. In contrast, PD-L1-positive tumors harbor stromal programs enriched in immune activation and controlled remodeling, consistent with an immunologically active microenvironment. This characterization, invisible to bulk transcriptomic analysis, suggests the potential role of tumor-associated macrophages in EMT and PD1/PD-L1 axis modulation [80,81,82,83]. This concept is supported by recent evidence showing that macrophage-derived extracellular vesicles can modulate immune cell function and shape inflammatory responses [84]. A landmark single-cell RNA sequencing study in glioblastoma by Hara et al. demonstrated that macrophages directly induce a transition of cancer cells into mesenchymal-like (MES-like) states associated with increased expression of a mesenchymal program in the macrophages themselves and with increased T-cell cytotoxicity, highlighting that the mesenchymal transition simultaneously reshapes both tumor cells and the immune microenvironment [85]. This finding is reinforced by pan-cancer TCGA analysis showing that 16 of 22 cancer types had significantly increased macrophage infiltration in EMT-high (mesenchymal) tumors (*p* < 0.001) [86]. Additionally, there is strong evidence supporting TAMs as the principal source of pro-angiogenic factors in the tumor microenvironment, functioning as the key effectors of the angiogenic switch [87,88]. Based on this rationale and our findings, these results suggest that targeting tumor-associated macrophages could offer therapeutic benefit, particularly in PD-L1-negative MSS CRC, by modulating EMT-associated immunosuppressive states and promoting features of host immune reactivation that may enhance responsiveness to immunotherapy [89]. This type of intervention has been recently described as a strategy to overcome EMT-associated immunosuppression in colorectal cancer by promoting M2-to-M1 macrophage polarization and restoring effective CD8^+^ T-cell infiltration [90].

Interestingly, 15% of cases in which the CMS status was assessed were classified as CMS1 despite being MMRp. This finding is consistent with the observations of Guinney et al., in which 24% of CMS1 tumors did not show MSI, as molecular subtypes are not defined by a single feature but rather by a combination of complex molecular characteristics [16]. These findings suggest that immune activation signatures can be observed in a subset of MSS tumors, highlighting the heterogeneous nature of the immune microenvironment in MSS colorectal cancer.

Although no other immune cell component showed prognostic relevance, the density and composition of TLSs were significantly associated with more abundant CD4+ and CD163+ cell infiltration and presented a borderline positive DFS association. TLSs have been reported as significantly associated with MSI and better prognosis in CRC cases [91]; however, their prognostic significance specifically in MSS cases has not been previously explored. Interestingly, when CMS1 cases were excluded from the analysis, TLSs were more frequently observed in CMS4 tumors, a finding that may be explored in future research as a potential stratification factor within this poor-prognosis molecular subtype.

The exploratory and observational design of this study and the absence of formal correction for multiple non-transcriptomic analyses represents a key limitation, as the findings have not been validated in independent cohorts or publicly available datasets. The absence of follow-up data in a subset of cases, together with the lack of adjustment for adjuvant therapy, may have introduced bias into the survival analyses. Accordingly, external validation and functional studies are needed to confirm the prognostic relevance of immune PD-L1 expression and to establish causality. To our knowledge, few studies have applied spatial transcriptomics specifically to MSS colorectal cancer; however, given the limited sample size in our study (n = 4), these findings should be considered exploratory, serving primarily as a proof of concept to support the bulk transcriptomic findings and to better characterize the role of the stromal compartment and macrophages in the EMT profile. Therefore, these findings require validation in larger independent cohorts.

## 5. Conclusions

In conclusion, our observations suggest a crosstalk between PD-L1 expression on immune cells and immune-activated versus mesenchymal-dominant states potentially mediated within tumor-associated macrophage-enriched stromal niches. Our findings provide a rationale to further investigate tumor-associated macrophages as potential therapeutic targets in PD-L1-negative MSS colorectal cancer, which should be explored in future functional studies.

## Figures and Tables

**Table 1:** Clinical and pathological features of the whole series (*n* = 254 patients).

	*n* (%)
**Gender**	
Male	133 (52.4)
Female	121 (47.6)
**Age (years)**	
≤65	127 (50.0)
>65	127 (50.0)
**Tumor location**	
Right colon	76 (29.9)
Left colon	148 (58.3)
Rectum	30 (11.8)
**Histological grade**	
Well/Moderately differentiated	200 (78.7)
Poorly differentiated	54 (21.3)
**Stage**	
I	35 (13.8)
II	120 (47.2)
III	99 (39.0)
**Lymphovascular invasion (LVI)**	
Absent	185 (72.8)
Present	69 (27.2)
**Perineural invasion (PNI)**	
Absent	206 (81.1)
Present	48 (18.9)
**Tumor budding (TB)**	
Low	137 (53.9)
High	117 (46.1)
**Tumor stroma (TS) content**	
Low	121 (47.6)
High	133 (52.4)
**Consensus molecular subtype (CMS)**	
CMS1	15 (15%)
CMS2	30 (30%)
CMS3	15 (15%)
CMS4	41 (41%)

**Table 2:** Association analysis of clinicopathological variables and PD-L1 expression in immune cells (≥1%).

Variable	Category	Odd Ratio	95% CI	*p*-Value
**Clinical stage**	I/II	3.53	2.11–6.24	***0.000***
	III	0.38	0.17–0.84	***0.017***
**Histological grade**	Low	1.32	0.86–4.44	*0.550*
	High	1.88	0.51–3.27	*0.120*
**PNI**	No	2.79	1.83–4.36	***0.000***
	Yes	0.36	0.13–1.01	***0.049***
**LVI**	No	3.05	1.91–5.04	***0.000***
	Yes	0.44	0.19–1.00	***0.050***
**VI**	No	2.66	1.75–4.13	***0.000***
	Yes	0.47	0.17–1.34	*0.149*
**pN**	N0	3.53	2.11–6.24	***0.000***
	N1	0.38	0.16–0.93	***0.032***
	N2	0.38	0.11–1.28	*0.108*
**pT**	T1	1.33	0.29–6.77	*0.706*
	T2	2.25	0.30–17.85	*0.424*
	T3	2.74	0.50–13.60	*0.215*
	T4	0.64	0.11–3.48	*0.607*
**r**	G1	3.21	2.06–5.18	***0.000***
	G2	0.26	0.07–0.93	***0.038***
	G3	0.18	0.04–0.64	***0.010***

## Data Availability

The bulk RNA sequencing data generated in this study have been deposited in the Gene Expression Omnibus (GEO) repository under accession number GSE319722 and are currently under embargo during peer review. The data will be made publicly available upon publication of the article. Processed data files and additional supporting materials are publicly available in Zenodo at https://doi.org/10.5281/zenodo.18620435 (accessed on 14 April 2025).

## Abbreviations

The following abbreviations are used in this manuscript:

CIs	Confidence intervals
CMSs	Consensus molecular subtypes
CRC	Colorectal cancer
DFS	Disease-free survival
EMT	Epithelial to mesenchymal transition
FDCs	Follicular dendritic cells
FFPE	Formalin-fixed paraffin embedded
HE	Hematoxylin and eosin
HRs	Hazard ratios
ICIs	Immune checkpoint inhibitors
IF	Invasive front
IHC	Immunohistochemistry
iTLSs	Immature tertiary lymphoid structures
LVI	Lymphovascular invasion
MMRp	Mismatch repair-proficient
MSI	Microsatellite instability
MSS-CRC	Microsatellite-stable colorectal cancer
mTLSs	Mature tertiary lymphoid structures
OR	Odds ratio
ORA	Overrepresentation analysis
pNs	Lymph nodes
PNI	Perineural invasion
pT	Depth of invasion
TB	Tumor budding
TC	Tumor center
TLSs	Tertiary lymphoid structures
TME	Tumor microenvironment
TS	Tumor stroma
